# IGF-1 contributes to the expansion of melanoma-initiating cells through an epithelial-mesenchymal transition process

**DOI:** 10.18632/oncotarget.12733

**Published:** 2016-10-18

**Authors:** Vincent Le Coz, Chaobin Zhu, Aurore Devocelle, Aimé Vazquez, Claude Boucheix, Sandy Azzi, Cindy Gallerne, Pierre Eid, Séverine Lecourt, Julien Giron-Michel

**Affiliations:** ^1^ INSERM UMRS 1197, Hôpital Paul Brousse, 94807 Villejuif Cedex, France; ^2^ INSERM UMRS 1193, Hôpital Paul Brousse, 94807 Villejuif Cedex, France; ^3^ Université Paris-Saclay, 91190, France

**Keywords:** IGF-1, melanoma-initiating cells, metastasis, EMT, chemoresistance

## Abstract

Melanoma is a particularly virulent human cancer, due to its resistance to conventional treatments and high frequency of metastasis. Melanomas contain a fraction of cells, the melanoma-initiating cells (MICs), responsible for tumor propagation and relapse. Identification of the molecular pathways supporting MICs is, therefore, vital for the development of targeted treatments. One factor produced by melanoma cells and their microenvironment, insulin-like growth factor-1 (IGF- 1), is linked to epithelial-mesenchymal transition (EMT) and stemness features in several cancers.

We evaluated the effect of IGF-1 on the phenotype and chemoresistance of B16-F10 cells. IGF-1 inhibition in these cells prevented malignant cell proliferation, migration and invasion, and lung colony formation in immunodeficient mice. IGF-1 downregulation also markedly inhibited EMT, with low levels of ZEB1 and mesenchymal markers (N-cadherin, CD44, CD29, CD105) associated with high levels of E-cadherin and MITF, the major regulator of melanocyte differentiation. IGF-1 inhibition greatly reduced stemness features, including the expression of key stem markers (SOX2, Oct-3/4, CD24 and CD133), and the functional characteristics of MICs (melanosphere formation, aldehyde dehydrogenase activity, side population). These features were associated with a high degree of sensitivity to mitoxantrone treatment.

In this study, we deciphered new connections between IGF-1 and stemness features and identified IGF-1 as instrumental for maintaining the MIC phenotype. The IGF1/IGF1-R nexus could be targeted for the development of more efficient anti-melanoma treatments. Blocking the IGF-1 pathway would improve the immune response, decrease the metastatic potential of tumor cells and sensitize melanoma cells to conventional treatments.

## INTRODUCTION

Melanoma is a particularly aggressive cancer responsible for a large proportion of skin cancer-related deaths. This tumor, arising from melanocytes, is notorious for its intrinsic resistance to chemotherapy, aggressive clinical behavior, and tendency to metastasize rapidly. There is currently no effective treatment for metastatic melanoma, and five-year survival does not exceed 15% in patients with metastatic disease [1].

There is a growing body of evidence to suggest that some of the cell types within the heterogeneous population constituting a melanoma display molecular and functional features similar to those of stem cells. These putative melanoma-initiating cells (MICs) have unlimited self-renewal and multilineage differentiation capabilities and can initiate and maintain tumor growth [2–8]. MICs have been identified with various stemness markers, including CD133, CD44, nestin, aldehyde dehydrogenase (ALDH), CD166, neural crest nerve growth factor receptor (CD271) and/or ATP-binding cassette (ABC) multidrug resistance transporters such as multidrug resistance-1 encoding P-glycoprotein (P-gp), ABCG2 and ABCB5. However, there is currently no single marker specific for MICs and the concept of tumor-initiating cells remains highly controversial. The MIC subset is rare in many solid tumors, but several studies have demonstrated that these cells may constitute a relatively large proportion, perhaps even the majority, of cells in certain aggressive tumors, such as breast cancers, glioblastomas and melanomas [9]. Indeed, MICs have been reported to account for 0.0001% to over 40% of the cells in melanomas [10–12]. The high degree of dispersal of these results reflects a high degree of dependence on the immune status of the mouse strains used for serial transplantation in animal models. The frequency of MICs has been shown to correlate with neoplastic progression, metastatic potential and poor prognosis in melanoma patients [3, 8]. Furthermore, MICs are thought to be resistant to both conventional chemotherapy agents and newly developed targeted drugs [13–14], and they have immunomodulatory properties favoring immune escape [15]. Recent studies have begun to explore ways of targeting MICs [16–18], but the molecular players involved in the maintenance of these cells have yet to be identified. Elucidation of the mechanisms and molecular intermediates favoring stemness features is a key area in cancer studies. There is growing evidence to suggest that the epithelial-mesenchymal transition (EMT) of tumor cells plays an important role in the acquisition of stem cell characteristics (self-renewal, tumorigenic potential, resistance to conventional therapies) [19–20]. During EMT, the cells discard their epithelial characteristics, including cell adhesion and polarity, reorganize their cytoskeleton and acquire a mesenchymal morphology and migratory phenotype. In this context, it has been shown that malignant melanocytes can be reprogrammed to differentiate into mesenchyme-like cells through a process similar to EMT during melanoma progression [21].

The production of growth factors by either the melanoma cells themselves or the tumor microenvironment is a critical event in the acquisition of a malignant phenotype. The local production of growth factors sustains cell proliferation and protects the melanoma against apoptosis, potentially accounting for its resistance to chemotherapy and observed immune escape. IGF-1 has been identified as a key driver of cell proliferation, in the development and growth of multiple tumors and in the prevention of apoptosis [22]. The biological actions of IGF-1 are mediated by the ligand-induced activation of the IGF-1 receptor (IGF-1R), a transmembrane tyrosine kinase linked to the Ras/Raf/mitogen-activated protein kinase and the phosphatidylinositol-3 kinase/protein kinase B/AKT signal-transduction cascades. Several recent studies have shown that IGF-1 also induces EMT and favors the development of stemness features in various tumor cells, partly accounting for the migratory properties, invasiveness and resistance to conventional treatments reported in previous studies [23–25]. However, the role of IGF-1 in melanoma metastasis remains unclear. We previously showed that IGF-1 mediated tumor immune escape by decreasing the immunogenicity of the murine melanoma B16-F0 cell line [26]. In the study described here, we investigated the effects of IGF-1 downregulation on the stemness features of murine melanoma B16-F10 cells and their tumorigenic potential in an NSG mouse model. The downregulation of IGF-1 expression in B16-F10 cells resulted in a lower proliferation potential, migration/invasion abilities and chemoresistance *in vitro*. These characteristics were associated with low levels of expression for EMT-related molecules and stemness markers, and weak tumorigenicity in mice. These findings indicate that IGF-1 plays a direct role in the intrinsic tumorigenic potential of metastatic tumor cells, promoting EMT and the development of stemness features in melanoma cells.

## RESULTS

### IGF-1 inhibition impairs the formation of B16-F10 melanoma cell colonies in the lungs of immunocompetent and immunodeficient mice

**Figure 1:**
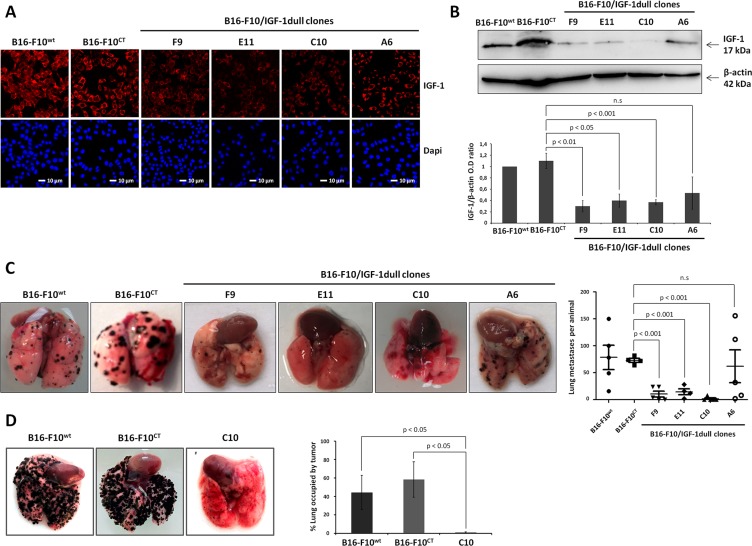
IGF-1 downregulation impairs lung colony formation by B16-F10 melanoma cells in immunocompetent and immunodeficient mice (**A**) IGF-1 levels were analyzed in four IGF-1dull clones (A6, C10, E11, F9), the control B16-F10^CT^ and the parental B16-F10^WT^ cells, by confocal microscopy. Red, IGF-1; Blue, nuclei stained with DAPI. The scale bar represents 10 μm. (**B**) IGF-1 levels in the various cell lines were analyzed by immunoblotting. β-actin was used as a loading control. Graphs show quantification of the immunoblot signals (IGF-1/β-actin). Data are represented as the mean ± standard error of mean (SEM) for three independent determinations. n.s., not significant, *p* < 0.05 *p* < 0.01, *p* < 0.001 versus B16-F10^CT^ cells. (**C–D**) Lung colony formation. Groups of C57BL/6 and NSG mice received 1 × 10^5^ cells via injection into the retro-orbital sinus (day 0). Fifteen days after inoculation with cells, the lungs were excised and nodule development was analyzed. Representative images of the lung nodules are shown. Horizontal bars represent the mean number of lung colonies ± SEM per C57BL/6 mouse. Clones E11 (14.5 ± 5.3 nodules), F9 (10.4 ± 5.6 nodules) and C10 (1.6 ± 1.6 nodules) had a significantly lower level of lung colony formation (*p* < 0.001, *n* = 5) than clone A6 (62 ± 30.6 nodules), B16-F10^CT^ (73.8 ± 19.2 nodules) and B16-F10^WT^ (73 ± 3.6 nodules) cells. n.s., not significant. In NSG mice, nodule development was quantified as the percentage of the lung occupied by tumors. Images of representative tumors are displayed for each *in vivo* experiment. The C10 clone formed lung colonies significantly less efficiently than B16-F10^CT^ and B16-F10^WT^ cells (1 ± 0.6% versus 58.3 ± 23 % and 44.1 ± 18.6% of the lung occupied by tumors, respectively, *p* < 0.05, *n* = 3).

We evaluated the impact of IGF-1 on melanoma, by first transfecting B16-F10 cells with an episomal IGF-1 antisense vector and selecting hygromycin-resistant clones (A6, C10, E11 and F9), in which we evaluated intracellular IGF-1 expression. Consistent with confocal microscopy analysis (Figure 1A), immunoblotting (Figure 1B) showed IGF-1 expression levels to be significantly lower in all clones (C10, E11, and F9), except A6, than in wild-type B16-F10 (B16-F10^WT^) cells and control B16-F10 (B16-F10^CT^) cells, a clone selected after transfection with an empty vector.

We then investigated the possible effects of IGF-1 depletion on the ability of B16-F10 cells to form colonies in the lung. We inoculated seven-week-old female C57BL/6 mice with the four engineered clones and the control clones (B16-F10^WT^ and B16-F10^CT^), by injecting 1 × 10^5^ cells (100 μL) intravenously into the retro-orbital sinus. The lungs were excised 15 days later, and lung colonies were counted under a dissecting microscope (Figure 1C). Tumor analysis revealed that B16-F10^CT^ cells produced similar numbers of lung nodules to B16-F10^WT^ cells, whereas the E11 and F9 clones formed significantly fewer colonies in the lungs. The most striking difference concerned clone C10, which produced few nodules. By contrast, clone A6 was not significantly affected. It produced larger amounts of IGF-1 than the other three IGF-1-dull. Similar experiments were carried out on immunodeficient NSG mice, to rule out the possibility of IGF-1-induced immune interference and to determine whether IGF-1 is involved in the intrinsic ability of B16-F10 to form lung colonies (Figure 1D). For these experiments, we used clone C10, which exhibits the lowest levels of IGF-1 and produces the smallest numbers of colonies in the lungs of C57BL/6 mice. Clone C10 cells also have lower levels of phosphorylated AKT and ERK-1/2 MAPK than B16-F10^CT^ and B16-F10^WT^ cells (Supplementary Figure S1A), confirming the attenuation of signaling via the major IGF-1-mediated pathways, the PI3K/AKT and MEK/ERK axis, in clone C10. IGF-1R levels remained unchanged (Supplementary Figure 1B). Fifteen days after the injection of cells into NSG mice, the lungs were excised and nodule development was quantified as the percentage of the lung occupied by tumor. As observed in immunocompetent recipient mice, the C10 clone generated smaller numbers of nodules than B16-F10^CT^ and B16-F10^WT^ cells, demonstrating a crucial role for IGF-1 in the control of the intrinsic ability of B16-F10 cells to form lung colonies.

### IGF-1 inhibition impairs the proliferation of B16-F10 melanoma cells

**Figure 2:**
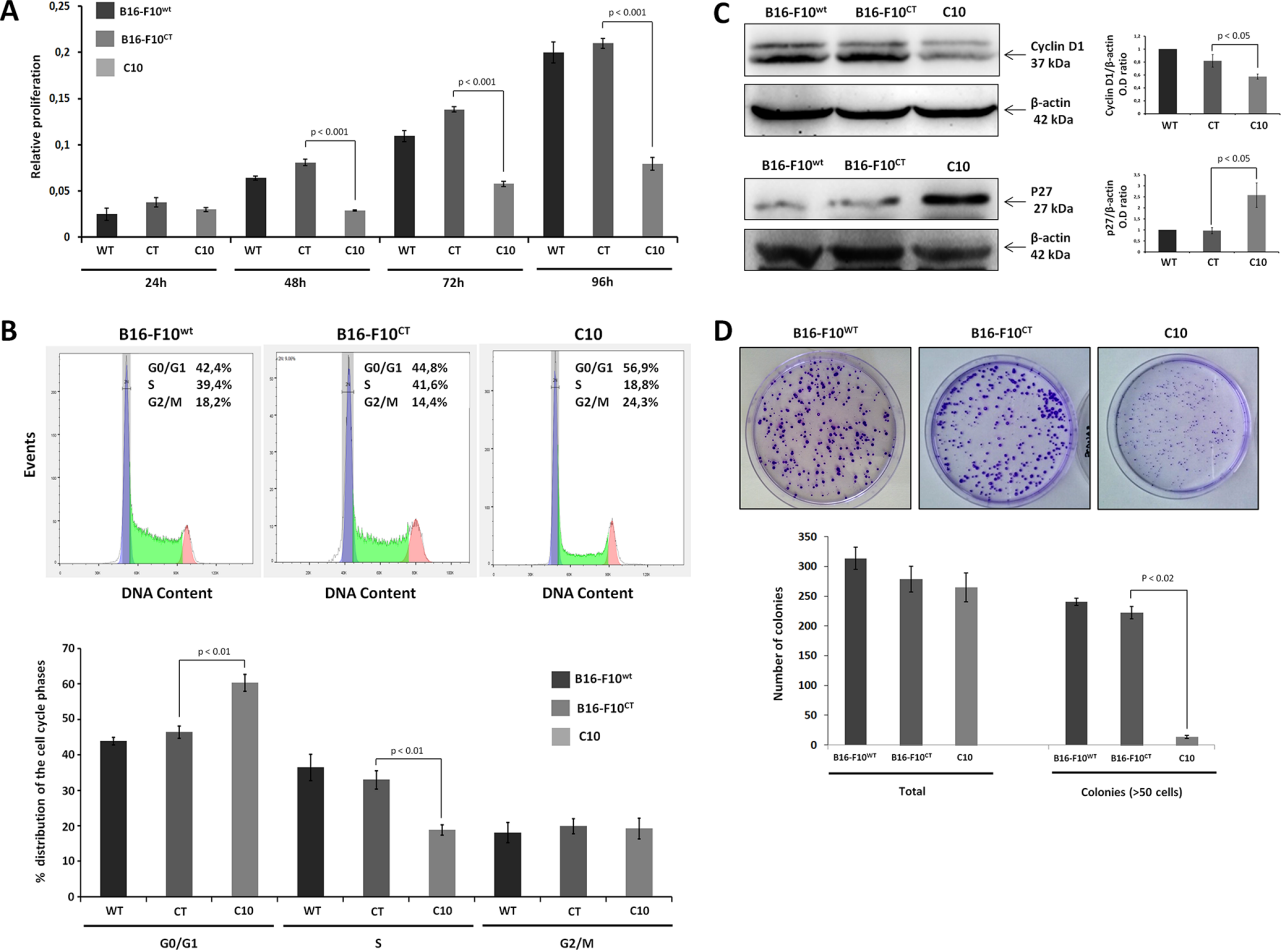
IGF-1 downregulation impairs cell proliferation (**A**) B16-F10 cell proliferation. B16-F10^WT^, B16-F10^CT^ and C10 cells were plated in 96-well plates and cell proliferation was assessed for 96 h in the MTT assay. The data presented are the mean (±SEM) of quadruplicate experiments. C10 had a lower proliferation rate than control cells, from 48 h onwards (0.08 ± 00.7 versus 0.002 ± 0.012 OD values, *p* < 0.001, *n* = 4). (**B**) Cell cycle analysis. After staining with propidium iodide, the distribution of the cells between the G0/G1, S and G2/M phases was determined by flow cytometry. *Top*, Representative cell cycle plots from one experiment are shown. The distribution and percentage of cells in the G0/G1, S and G2/M phases of the cell cycle are indicated at the top right of each histogram. *Bottom*, Data are represented as means ± SEM. IGF-1 downregulation resulted in a lower percentage of cells in S-phase (18.9±1.4%) for C10 cells than for B16-F10^WT^ (36.5 ± 3.7%) and B16-F10^CT^ cells (33.0 ± 2.5%, *p* < 0.001, *n* = 4), and a higher percentage of cells in G0/G1 phase in C10 cells (60.4% +/–2.3) than in B16-F10^WT^ (43.9 ± 1.0%) and B16-F10^CT^ cells (46.4 ± 1.7%, *p* < 0.001, *n* = 4). (**C**) Quantification of cyclin D1 and p27 protein levels in B16-F10^WT^, B16-F10^CT^ and C10 cells. Graphs represent the quantification of immunoblot signals, corresponding to the cyclin D1/β-actin and p27/β-actin ratio. Data are represented as means ± SEM for three independent determinations. (**D**) Clonogenic assay. B16-F10^WT^, B16-F10^CT^ and C10 cells were assessed for clonogenic potential. The data presented are the means (±SEM) of quintuplet experiments. C10 colonies were significantly smaller than the colonies for control and parental cells (14 ± 2 colonies with a diameter > 75 μm for the C10 clone versus 241 ± 6 and 223 ± 10 for B16-F10^WT^ and B16-F10^CT^, respectively, *p* < 0.02, *n* = 5).

IGF-1 plays a key role in the development and progression of various cancers. However, little is known about the molecular mechanisms underlying IGF-1-mediated metastatic potential in melanoma. IGF-1 has clearly been shown to be a potent mitogen. We therefore used the MTT assay to determine whether decreases in IGF-1 levels affected the proliferation of B16-F10 cells (Figure 2A). From 48 h onwards, C10 cells had a lower proliferation rate than B16-F10^CT^ cells. There was no significant difference between B16-F10^WT^ and B16-F10^CT^ cells. The mechanism by which IGF-1 stimulates cell proliferation is well documented. Indeed, IGF-1 is known to activate the cell cycle by inducing the expression of cyclins and CDKs, facilitating the G1/S transition [27]. We used flow cytometry to determine whether the downregulation of IGF-1 affected cell cycle progression in B16-F10 cells. We determined the distribution of C10, B16-F10^CT^ and B16-F10^WT^ cells between the G0/G1, S and G2/M phases (Figure 2B). Lower levels of IGF-1 were associated with a lower percentage of cells in S-phase than in the parental and control cells, with a higher percentage of cells in the G0/G1 phase. Thus, as expected, IGF-1 promotes G1/S cell cycle progression and proliferation in melanoma cells. This finding was further confirmed by immunoblotting (Figure 2C), which showed p27 (CDK inhibitor) levels to be higher, and cyclin D1 levels to be lower in the C10 clone. The lower proliferation rate of the C10 clone was also confirmed by clonogenic assays (Figure 2D). The total number of colonies was similar in all conditions, but the colonies of the IGF-1-dull C10 clone were significantly smaller than those formed by B16-F10^CT^ and B16-F10^WT^ cells.

### IGF-1 drives EMT and promotes cell migration and the invasive potential of B16-F10 cells

**Figure 3:**
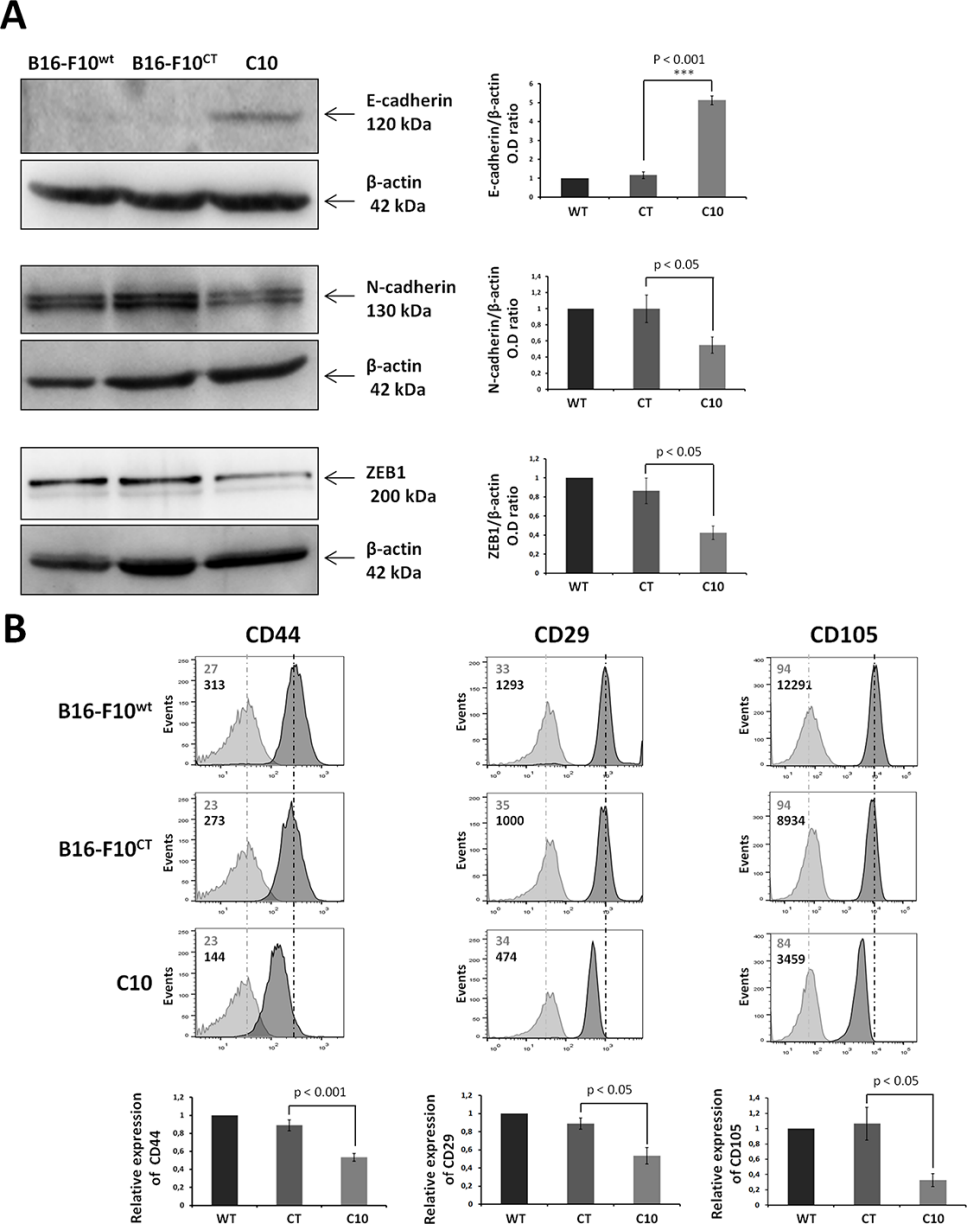
IGF-1 downregulation promotes mesenchymal-to-epithelial transition (**A**) Western blot analysis of an epithelial marker (E-cadherin), a mesenchymal marker (N-cadherin), and an EMT regulator (ZEB1) in C10, B16-F10^CT^ and B16-F10^WT^ cells. β-actin was used as the loading control. Graphs represent the quantification of the immunoblot signals corresponding to the protein of interest/β-actin ratio. Data are represented as means ± SEM for three independent determinations, *p* < 0.05, *p* < 0.001 versus B16-F10^CT^ cells. (**B**) Flow cytometry analysis of surface expression of the mesenchymal markers CD44, CD29 and CD105. The dark-colored bars correspond to cells incubated with the antigen-specific antibody, and gray bars correspond to cells incubated with the isotype-matched control antibody. Mean fluorescence intensity values for each marker are shown at the top left of each histogram. Each panel corresponds to three independent experiments with similar results, *p* < 0.05, *p* < 0.001 versus B16-F10^CT^ cells.

Epithelial-mesenchymal transition is a key step in tumor invasion and progression. We investigated the role of IGF-1 in EMT regulation, by analyzing the levels of E-cadherin, N-cadherin and ZEB1 in B16-F10 cells. On immunoblots, the downregulation of IGF-1 was associated with higher levels of the epithelial marker E-cadherin, and lower levels of the mesenchymal marker N-cadherin (Figure 3A). Levels of zinc finger E-box-binding protein 1 (ZEB1), a major driver of the EMT process in melanoma cells [28–30], were also significantly lower in the C10 clone (Figure 3A). These results were supported by flow cytometry analysis, which showed markedly lower levels of the mesenchymal markers CD29, CD44 and CD105 in the C10 clone than in control and parental cells (Figure 3B).

**Figure 4:**
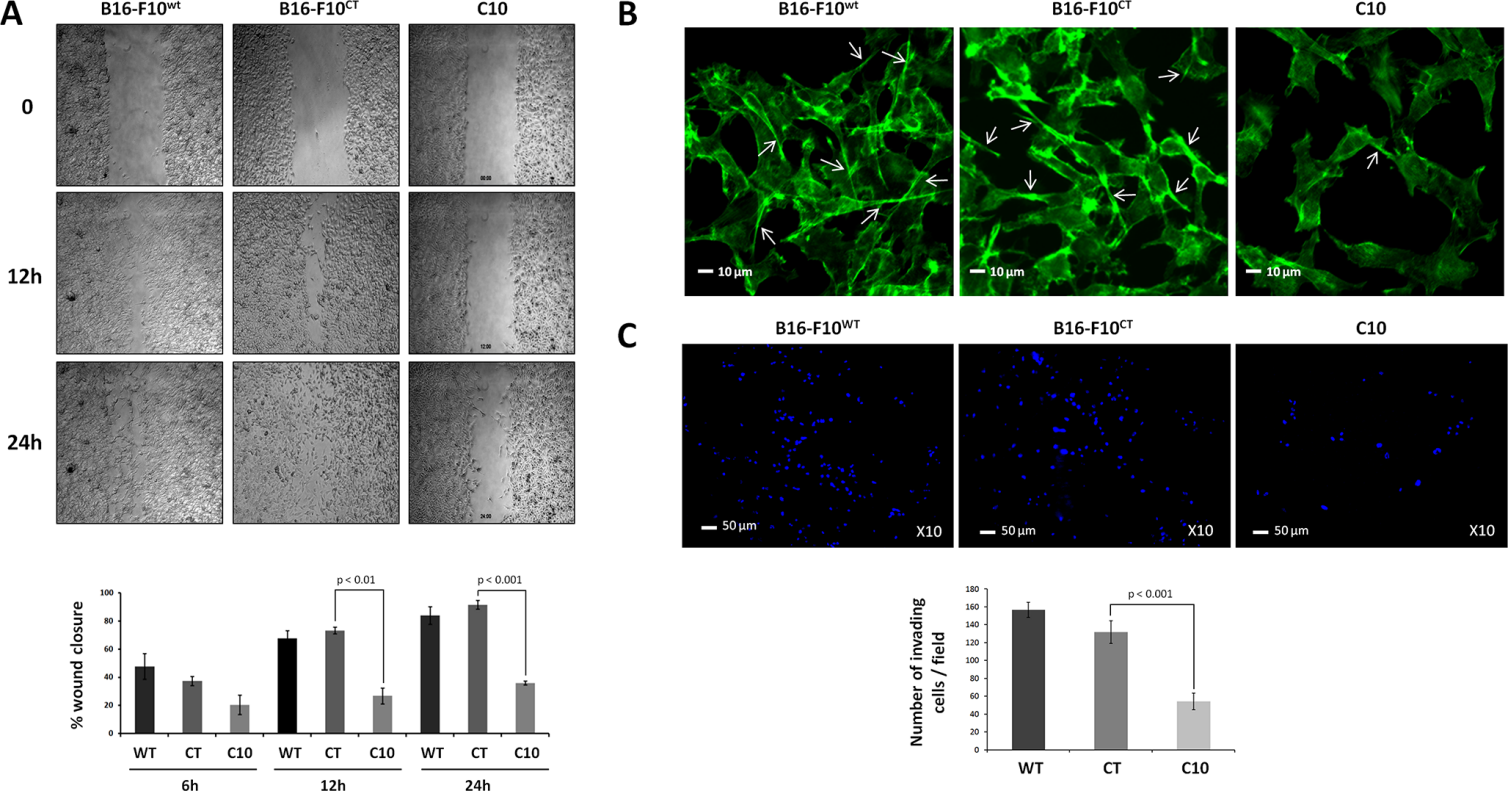
IGF-1 downregulation decreases cell invasion and migration (**A**) Scratchwound healing assay. Cells were grown to confluence and then used for wound healing migration assays. Artificial wounds were made in confluent monolayers of C10, B16-F10^CT^ or B16-F10^WT^ cells. *Top*, Migration of melanoma cells towards the wound, photographed after 6 h, 12 h and 24 h. The top panel shows one of three independent experiments. *Bottom*, Data are shown as means ± SEM. No significant differences in migratory activity were observed between B16-F10^WT^ and B16-F10^CT^ cells, with 67.7 ± 5.4% and 73.3 ± 2.4%, respectively, of the area displaying recovery at 12 h and 83.9 ± 6.3% and 91.6 ± 3.1%, respectively, of the area displaying recovery at 24 h. However, C10 clones displayed lower levels of migratory activity, with only 26.7 ± 5.5% (*p* < 0.01, *n* = 3) and 35.9 ± 1.4% (*p* < 0.001, *n* = 3) of recovery at 12 h and 24 h, respectively. (**B**) F-actin expression was analyzed by fluorescence microscopy with phalloidin (green) as a marker of membrane protrusions. The scale bar represents 10 μm. White arrowheads indicate the pseudopods. Each experiment was performed three times. (**C**) Invasion assay. C10, B16-F10^CT^ and B16-F10^WT^ cells were cultured in Matrigel Transwells. Their invasive potential was analyzed 96 h later, by fluorescence microscopy. The nuclei are stained blue with DAPI. *Top,* Representative images of three independent experiments are shown. The scale bar represents 50 μm. *Bottom,* Data are shown as means ± SEM. C10 clones have a lower invasion capacity (54.2 ± 9.2 cells per field) than parental (156.6 ± 8.3 cells per field) and control cells (131.6 ± 12.6 cells per field, *p* < 0.001, *n* = 3).

As EMT promotes invasiveness in cancer cells [31], we then investigated whether the EMT triggered by IGF-1 affected the migration and invasion properties of B16-F10 cells. We first examined the effect of lowering IGF-1 levels on the migration of B16-F10 cells in a scratchwound healing assay. Clone C10 displayed lower levels of migration than parental B16-F10^WT^ and control B16-F10^CT^ cells (Figure 4A). Cell migration through the extracellular matrix (ECM) requires the formation of cell protrusions containing filamentous actin (F-actin), which recognize the external environment. Immunofluorescence analysis of F-actin with phalloidin (Figure 4B) revealed the presence of protrusions emanating from B16-F10^WT^ and B16-F10^CT^ cells. By contrast, C10 cells had much smaller numbers of membrane protrusion, partly accounting for the lower migratory capacity of these cells. We then analyzed the invasive capacity of IGF-1dull C10 cells. In Matrigel invasion assays *in vitro*, IGF-1 silencing resulted in significantly lower levels of invasion by B16-F10 cells than by parental and control cells (Figure 4C). Overall, these data confirm that IGF-1 enhances EMT and promotes cell migration and invasion.

### IGF-1 maintains MIC features in the B16-F10 cell line, throughout EMT

**Figure 5:**
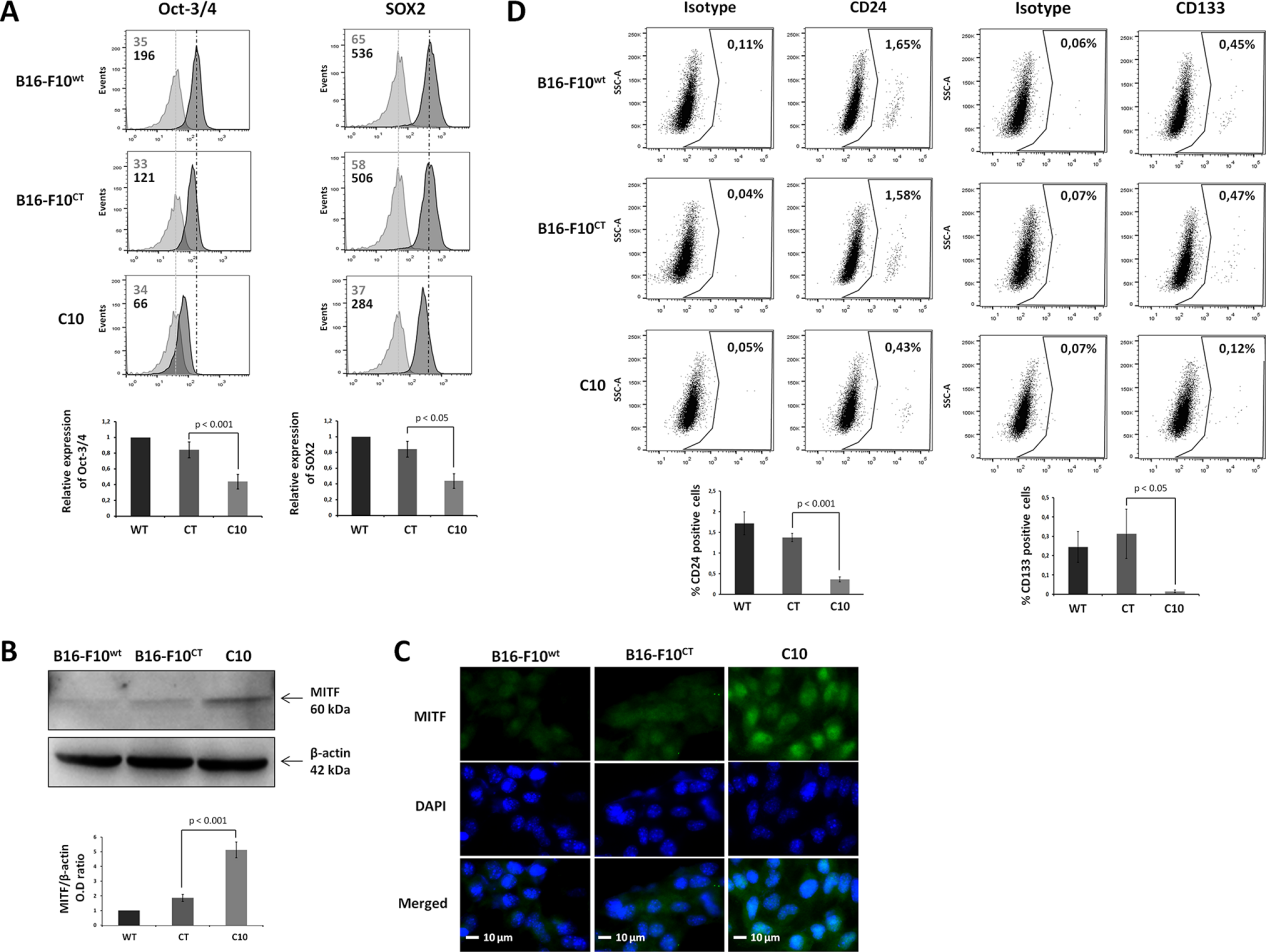
IGF-1 downregulation decreases the expression of cancer stem-like and pluripotency markers in B16-F10 melanoma cells (**A**) Flow cytometry analysis of the canonical self-renewal transcription factors Oct-3/4 and SOX2 in the C10 clone, B16-F10^CT^ and B16-F10^WT^ cells. *Top,* Dark-colored bars correspond to cells incubated with the antigen-specific antibody, and gray bars correspond to cells incubated with the isotype-matched control antibody. Mean fluorescence intensity values for each marker are shown at the top left of each histogram. All panels represent at least four independent experiments showing similar results. *Bottom*, Data are represented as means ± SEM for three independent determinations, *p* < 0.05, *p* < 0.01 versus B16-F10^CT^ cells. (**B**) Western-blot analysis for MITF in C10, B16-F10^CT^ and B16-F10^WT^ cell lysates. β-actin was used as the loading control. *Bottom*, Graphs show the quantification of three immunoblot signals corresponding to the protein of interest/β-actin ratio, *p* < 0.01 versus B16-F10^CT^ cells. (**C**) Analysis of MITF expression (green) by fluorescence microscopy. Blue, nuclei stained with DAPI. The scale bar represents 10 μm. The panel depicts one of three independent experiments. (**D**) CD24 and CD133 cell surface markers were analyzed by flow cytometry. Top, Panels represent at least four independent experiments with similar results. The percentages of CD24- and CD133-positive cells are shown at the top right of each histogram. *Bottom*, Data are represented as means ± SEM for three independent determinations, *p* < 0.05, *p* < 0.001 versus B16-F10^CT^ cells.

Epithelial-mesenchymal transition is known to enhance stemness features in several tumor cell models. However, the role of IGF-1 in the stem cell phenotype of some melanoma cells remains to be determined. We investigated whether IGF-1 was required to maintain the stem-like cell phenotype in B16-F10 cells, by analyzing the expression of biomarkers classically used for MIC identification [32–33]. Levels of the pluripotent transcription factors Oct-3/4 and Sox2 were markedly low in the C10 clone (Figure 5A). By contrast, immunoblotting results (Figure 5B) revealed that IGF-1 downregulation was associated with an increase in the levels of microphthalmia-associated transcription factor (MITF), a master regulator of melanocyte differentiation controlling the differentiation of MICs. This finding was confirmed by immunofluorescence analysis (Figure 5C), which showed that MITF expression was mostly nuclear and stronger in the C10 clone than in parental and control cells. Moreover, the percentage of CD24^+^ and CD133^+^ cells in the B16-F10 population was significantly lower after IGF-1 inhibition (Figure 5D), demonstrating the key role of IGF-1 in the maintenance of stemness features in the most primitive MICs (CD44^+^CD133^+^CD24^+^), which make up less than 1% of the B16-F10 population [33]. EMT (CD29, CD44) and stemness (Oct-3/4, Sox2) marker levels were decreased by IFG-1R neutralization in B16-F10^WT^ and B16-F10^CT^ cells (Supplementary Figure S2).

**Figure 6:**
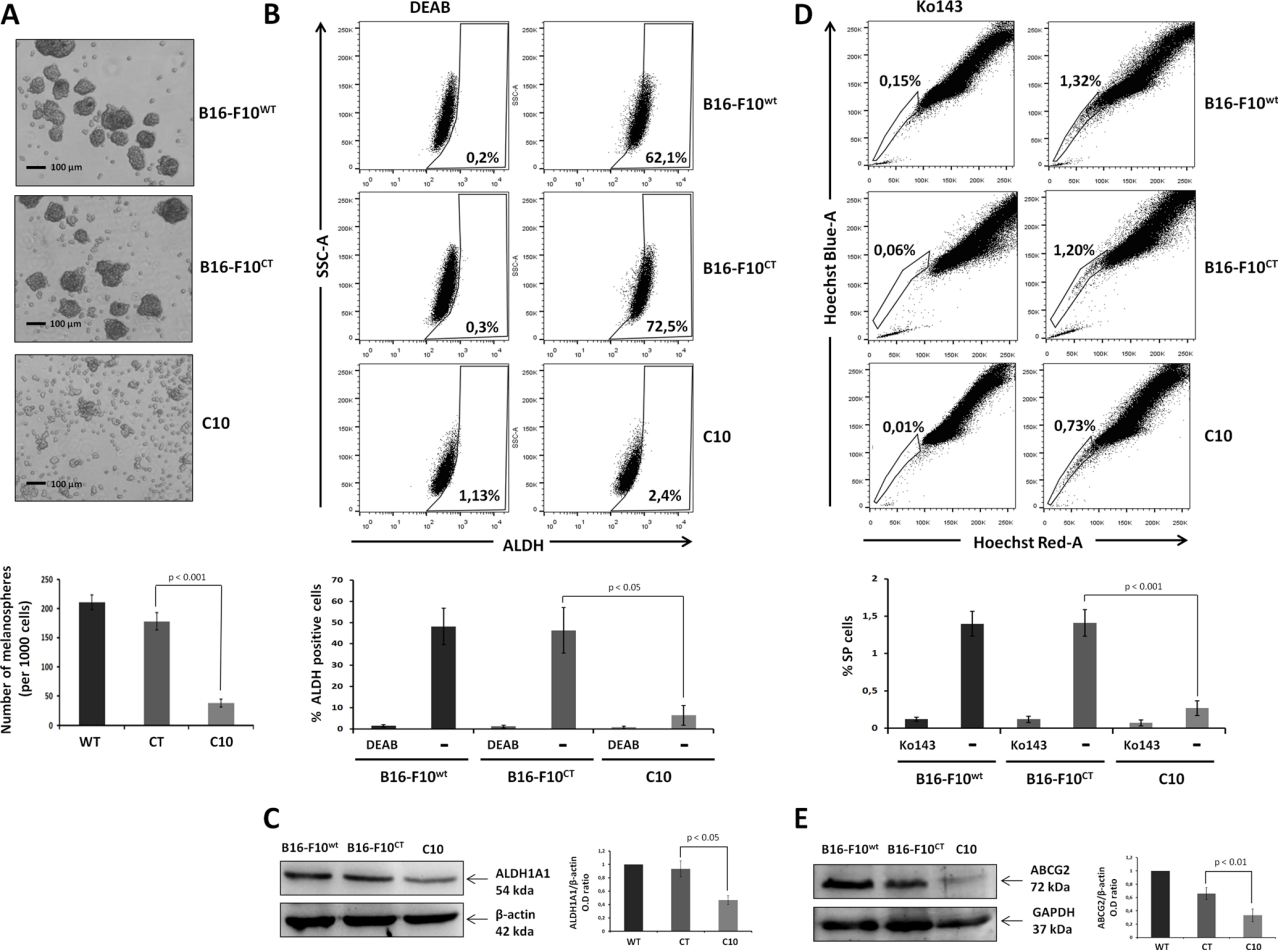
IGF-1 knockdown affects stemness properties and MIC behavior (**A**) Melanosphere formation assay. We assessed the ability of parental B16-F10 cells to produce tumor spheres, by suspending 1x10^3^ cells in a serum-free medium and incubating them at 37°C for 7 days. The total number of spheres produced was compared between groups. The scale bar represents 50 μm. *Top,* Independent experiments were performed three times and a representative experiment is shown. *Bottom,* Data are shown as means ± SEM. The melanospheres for the IGF-1-knockdown clone (C10) were significantly smaller and fewer (38.3 ± 6.7 spheres per 1000 cells) than those formed by B16-F10^WT^ (210.6 ± 12.9 spheres per 1000 cells) and B16-F10^CT^(178.3 ± 14.6 spheres per 1000 cells, *p* < 0.001, *n* = 3) cells. (**B**) ALDH activity. ALDH1A1 activity was analyzed in B16-F10^WT^, B16-F10^CT^ and C10 cells, by flow cytometry with the Aldefluor reagent kit. ALDH1A1 activity was normalized by taking measurements in the presence and absence of the ALDH1-specific inhibitor, diethylamino-benzaldehyde (DEAB: 10 μM). The top panel depicts one of four experiments. The percentage ALDH-positive cells is shown at the bottom right of each histogram. *Bottom,* Data are shown as means ± SEM. ALDH activity was much weaker in C10 clones (6.5 ± 9.2%) than in B16-F10^WT^ and B16-F10^CT^ cells, which had high levels of ALDH activity (48.2 ± 19.2% and 46.4 ± 23.9%, *p* < 0.05, *n*=4 respectively). (**C**) ALDH1A1 protein levels, as determined by immunoblotting. β-actin was used as the loading control. Graphs represent the quantification of immunoblot signals, corresponding to the ALDH1A1/β-actin ratio. The data shown are the means ± SEM of three independent determinations, *p* < 0.05. (**D**) Side population (SP) analysis. C10, B16-F10^CT^ and B16-F10^WT^ cells were stained with Hoechst 33342 dye in the presence and absence of Ko143 (1 μM), and the SP was analyzed by flow cytometry. *Top,* Data from at least five independent experiments with similar results. Percentages of SP cells are shown at the top left of each histogram. *Bottom,* Data are shown as means ± SEM. SP cells represent 1.4 ± 0.4% of all cells for B16-F10^WT^ and B16-F10^CT^ cells, but only 0.3 ± 0.2% of total C10 clone cells (*p* < 0.01, *n* = 5). (**E**) Analysis of ABCG2 expression in cell lysates of C10, B16-F10^CT^ and B16-F10^WT^. β-actin was used as the loading control. *Top,* Figures are representative of three independent experiments yielding similar results. *Bottom,* Data are shown as means ± SEM, *p* < 0.01 versus B16-F10^CT^ cells.

As MICs cannot be identified on the basis of universal cancer stem cell markers alone, the analysis of Oct-3/4, SOX2, CD133 and CD24 levels must be associated with behavioral bioassays (spheroid formation, side population cells and ALDH activity), in evaluations of the role of IGF-1 in stemness in melanoma. We investigated whether the modulation of IGF-1 levels affected the behavior of melanospheres, which are enriched in melanoma cells with tumor-initiating properties [33]. IGF-1dull melanospheres were significantly smaller and fewer in number than parental and control melanospheres (Figure 6A). We then analyzed the activity of the detoxifying iso-enzyme aldehyde dehydrogenase (ALDH1A1), which is widely considered to be a feature of stemness [34]. Indeed, the levels and activity of this enzyme could potentially be used to identify stem-like cells in melanoma tumors [2, 35]. We found that ALDH activity was abolished in the C10 clone, whereas it was high in parental and control cells, which had high levels of ALDH activity (Figure 6B). Consistent with the loss of ALDH activity, immunoblotting revealed the presence of lower levels of ALDH1A1 protein in the C10 clone (Figure 6C). We then checked the concordance between stemness markers and bioactivity assays, by evaluating the proportion of side population (SP) in the various B16-F10 cell cultures on the basis of extrusion of the Hoechst 33342 dye via the BCRP1/ABCG2 drug transporter. The SP fraction, identified in many solid tumors and tumor cell lines, including B16-F10 [36], is thought to correspond to a population of cells enriched in stem cells, which are more tumorigenic than other cancer cells. We stained C10, B16F10^WT^, and B16-F10^CT^ cells with Hoechst 33342 dye in the presence or absence of the ABCG2 transporter inhibitor Ko143 and carried out flow cytometry analysis. SP cells accounted for 1.4 ± 0.4% of both B16-F10^WT^ and B16-F10^CT^ cells, but only 0.3 ± 0.2% of total cells for the C10 clone (Figure 6D). The SP fraction of B16-F10^WT^ and B16-F10^CT^ cells was decreased after IGF-1R neutralization (Supplementary Figure S3A), consistent with the findings for the C10 clone. We investigated whether the loss of the SP fraction was associated with ABCG2 protein levels or activity, by immunoblotting to determine ABCG2 levels. ABCG2 levels were markedly lower in the C10 clone than in parental and control cells (Figure 6E). These data suggest that IGF-1 plays a crucial role in promoting the side population phenotype of B16-F10 cells.

### IGF-1 downregulation sensitizes B16-F10 cells to mitoxantrone

**Figure 7:**
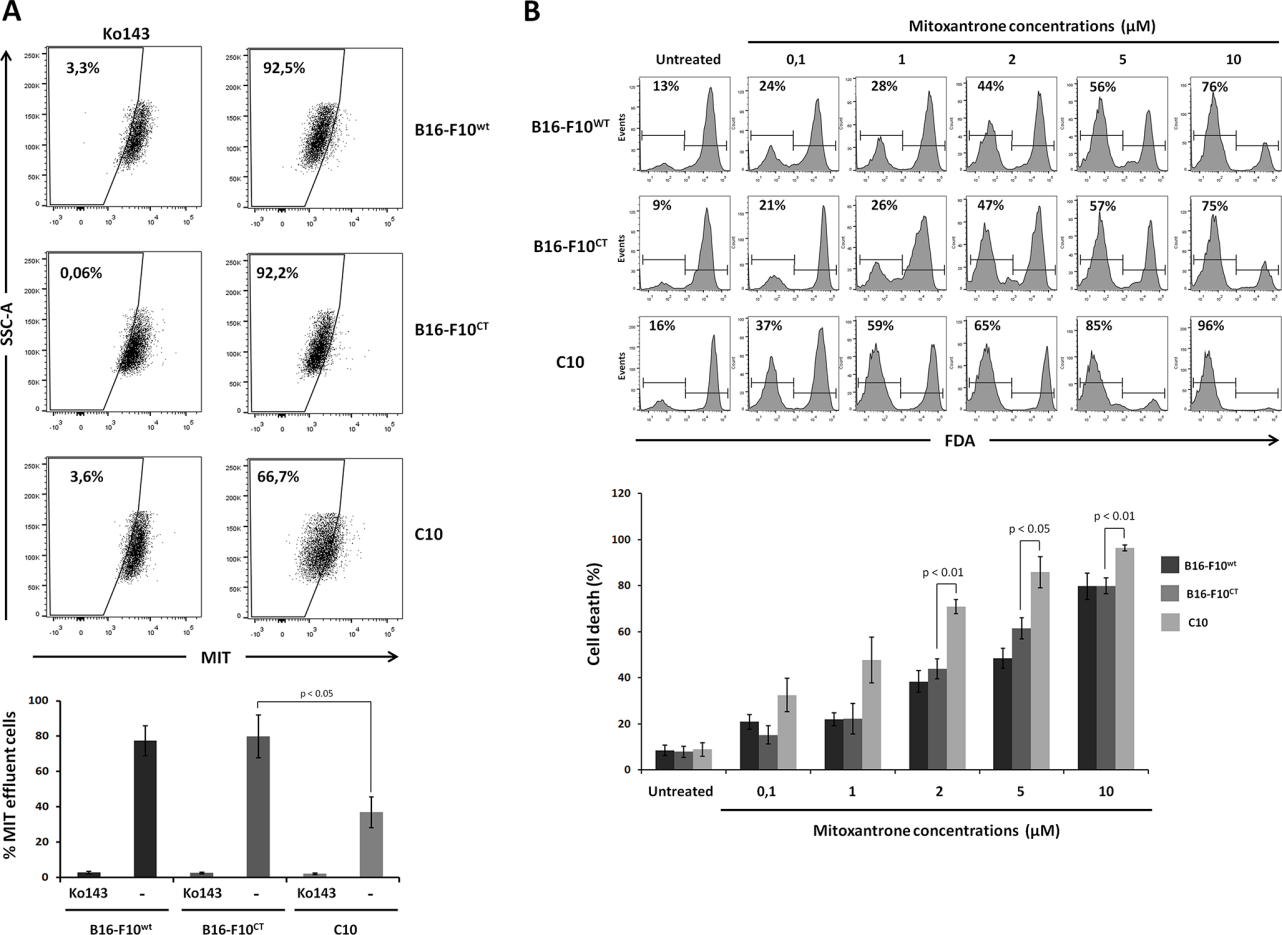
IGF-1 downregulation sensitizes B16-F10 cells to mitoxantrone (**A**) Analysis of the intracellular accumulation of MIT in B16-F10^WT^, B16-F10^CT^ and C10 cells. Mitoxantrone efflux and ABCG2 activity were determined by flow cytometry measurements of mitoxantrone accumulation in the presence and absence of the ABCG2 inhibitor Ko143 (1 μM). *Top,* Seven independent experiments were performed, and the results of a single representative experiment are shown here. Percentages of cells displaying MIT efflux are shown at the top left of each histogram. *Bottom,* Data are shown as means ± SEM. C10 cells were significantly less able to exclude MIT (36.9 ± 9.0%) than B16-F10^WT^ and B16-F10^CT^ cells (77.5 ± 21.2% and 79.9 ± 15.1%, respectively, *p* < 0.05, *n* = 7). (**B**) Cell viability. Cells were treated with different concentrations of mitoxantrone (0.1 to 10 μM) and cell viability was determined at 48 h, in the FDA assay. *Top* The panel depicts one of four experiments. Percentages of FDA-negative (dead) cells are shown at the top left of each histogram. *Bottom,* Data are shown as means ± SEM. B16-F10^WT^ and B16-F10^CT^ cells were consistently found to be more resistant to MIT than the C10 clone, from the dose of 2 μM MIT upwards, with 70.8 ± 3.0% cell death for the C10 clone versus 38.3 ± 4.7% and 43.8 ± 4.25% cell death for B16-F10^WT^ and B16-F10^CT^ cells, respectively, *p* < 0.01, *n* = 4.

We investigated whether ABCG2 protein levels reflected drug extrusion capacity, by determining intracellular levels of the anthraquinone anticancer drug mitoxantrone (MIT) in the different cell lines. MIT efflux and ABCG2 activity were determined by measuring MIT accumulation in the presence and absence of the ABCG2 inhibitor Ko143. Flow cytometry (Figure 7A) showed that intracellular MIT accumulation was increased by treating B16-F10^WT^cells with Ko143, confirming that dependence of MIT efflux on ABCG2. Moreover, MIT extrusion was significantly weaker for the C10 clone than for B16-F10^WT^ and B16-F10^CT^ cells. Intracellular MIT accumulation in B16-F10^WT^ and B16-F10^CT^ cells increased to levels similar to those observed in the IGF-1dull clone, following IGF-1R neutralization (Supplementary Figure S3B). Overall, these data indicate that IGF-1 governs drug efflux pump activity.

Given the close association between drug efflux and drug resistance, we assessed the sensitivity of the C10 clone to MIT. Cells were treated with various concentrations of MIT (0.1 to 10 μM) for 48 hours and were then subjected to a viability assay based on the fluorescent probe fluorescein diacetate (FDA). A quantitative analysis of living cells showed that MIT increased cell death in a dose-dependent manner, for all three cell lines (Figure 7B). B16-F10^WT^ and B16-F10^CT^ cells were found to be more resistant to MIT than the C10 clone, for concentrations exceeding 2 μM. As expected, the addition of the Ko143 inhibitor to the culture medium greatly decreased cell death rates in B16-F10^CT^ cells treated with 2 μM MIT, whereas it had no significant impact on C10 cell death (Supplementary Figure S4).

Overall these results are consistent with a role for IGF-1 as major regulator of ABCG2 pump activity and resistance to mitoxantrone in B16-F10 cells.

## DISCUSSION

In summary, we report here that IGF-1 is a key player in melanoma tumorigenicity and response to anti-tumor therapies. Indeed, we found that IGF-1 was involved in the intrinsic tumorigenic potential of metastatic tumor cells. It also triggered EMT in B16-F10 cells. This process is associated with a highly migratory and invasive phenotype of tumor cells. Finally, we show that IGF-1 favors MIC features in melanoma and governs the tumor response to conventional therapies.

The IGF axis has been identified as one of the molecular networks involved in the formation, progression and metastatic spread of many types of cancer. Epidemiological evidence suggests that high levels of circulating IGF-1 and IGF-1R expression are associated with a higher risk of several common cancers, including melanoma [37]. Moreover, high levels of IGF-1R expression are associated with poorer survival in patients with uveal melanoma [38]. This is not surprising, given the role of IGF-1 in several biological processes essential for malignancy, including cell growth, DNA repair, and survival, and in immune escape. There is also evidence supporting the involvement of IGF-1 in metastasis mechanisms, by promoting the acquisition of stem cell features. Cancer-initiating cells (CICs) constitute a minor subpopulation of undifferentiated cells responsible for sustaining and renewing the tumor. In particular, CICs have the characteristics of cancer cells that have undergone EMT and contribute to therapeutic resistance. Direct links between activated IGF-1R signaling and the acquisition of stem cell- and EMT-associated properties were recently reported in the CICs of several tumors, including lung adenocarcinoma [39–40], hepatocellular carcinoma [41], colon carcinoma [42], glioma [24–25] and breast cancer [43]. Consistent with the presence of CICs, or melanoma-initiating cells (MICs), in melanoma, and the role of IGF-1 in mediating EMT, chemoresistance, and stemness, we hypothesized that IGF-1 was involved in the acquisition of stem cells, promoting treatment resistance and melanoma cell propagation. We show that, in addition to its role in immune escape [26], IGF-1 affects cell migration and invasion, and promotes the MIC phenotype through EMT.

Various cellular growth factors elicit EMT and activate PI3K/AKT, a major IGF-1 signaling pathway, and a key component driving EMT. Indeed, AKT downregulates E-cadherin expression and promotes EMT-like transitions and invasiveness in carcinoma cells, by inducing the zinc-finger transcription factor snail [44]. Our results show that IGF-1 plays a critical role in EMT in B16-F10 cells. Indeed, IGF-1 downregulation resulted in morphological changes, an upregulation of epithelial marker expression, and a downregulation of mesenchymal markers (N-cadherin, CD29, CD44 and CD105) and ZEB1, which is essential for EMT induction in B16-F10 cells [28–30]. Indeed, one of the roles of ZEB1 is to regulate the expression of E-cadherin, a key molecule in the maintenance of epithelial cell characteristics [45], as its loss may enhance the invasive and metastatic behavior of melanoma cells [46]. We found that IGF-1 knockdown inhibited cell migration and invasion, together with pulmonary nodule formation *in vivo* in NSG mice. Many of the cell surface markers commonly used to define MICs, such as CD29, CD44, and CD24, are receptors for extracellular matrix components, or promote integrin-mediated binding to ECM components. A decrease in the expression of these mesenchymal markers and F-actin in IGF-1dull clones (C10) may partly account for the loss of invasive potential observed in B16-F10 cells.

High levels of EMT gene expression were observed in MICs, suggesting an overlap between the genetic signature and EMT [28, 47]. However, the links between EMT, IGF-1 and the tumorigenic potential of MICs have yet to be fully elucidated. Indeed, the molecular mechanisms underlying IGF-1-mediated stemness remain largely unknown in melanoma. We show here that IGF-1 is a key factor for the maintenance of MIC features throughout EMT in B16-F10 cells. Indeed, IGF-1 decreases the expression of microphthalmia-associated transcription factor (MITF) and upregulates the expression of stemness markers (Oct-3/4, SOX2) and of CD133 and CD24, consistent with IGF-1 being instrumental in the preservation of the rare CD44^+^CD133^+^CD24^+^ cell subset, accounting for less than 1% of the B16-F10 population, and potentially corresponding to the most primitive MICs [33]. IGF-1 downregulation in B16-F10 cells also led to a loss of MIC functions, including the ability to form melanospheres, the side population and ALDH activity. High levels of ALDH activity are a hallmark of MICs in melanoma. Indeed, ALDH1A1 has been associated with the tumor-initiating cell phenotype in human melanoma [2], and its levels and activity could potentially be used to identify stem-like cells in melanoma tumors [2, 35]. ALDH1A1 knockdown has been shown to decrease the pro-tumorigenic and pro-metastatic advantage of melanoma cells lacking dioxin receptor expression, decreasing not only their migration and invasion potentials, but also the percentage of CD133^+^CD29^+^CD44^+^ cells, melanosphere size and expression of the pluripotency marker SOX2 [32]. The shRNA-mediated knockdown of ALDH1A expression sensitizes melanoma cells to chemotherapy and affects melanoma tumor growth [34]. Like IGF-1 downregulation, IGF-1R neutralization led to a loss of MIC features in B16-F10 cells. However, no synergic effect of IGF-1R neutralization was observed in the IGF-1dull clone, suggesting the possible involvement of other regulatory factors in melanoma stemness behavior.

Metastatic melanoma is highly chemoresistant. Approximately 40–50% of melanomas harbor activating mutations of the RAS/RAF axis, including the V600E BRAF mutation in an oncogene known to be critical for melanoma proliferation and survival through its role in activating the RAF/MEK/ERK pathway [48]. The outlook has improved significantly following the development of novel forms of immunotherapy (e.g. anti-cytotoxic T-lymphocyte antigen 4 (CTLA-4) and anti-programmed death 1 (PD-1) antibodies) and mutant BRAF inhibitors, which have increased overall survival [49–50]. However, responses to mutant BRAF inhibitors are often brief, and resistance rapidly emerges [51–52]. Further studies are therefore required, on new novel therapeutic molecules, delivery systems and combination therapies for melanoma. The targeting of IGF-1 in melanoma appears promising for novel anti-cancer strategies, as IGF-1 favors EMT, which seems to be intimately linked to tumor cell stemness and resistance. In addition, many studies have shown that IGF-1R depletion reduces the survival of BRAF-mutant and wild-type melanoma cells, and increases their chemosensitivity [53–54]. Acquired resistance to BRAF inhibitors in melanoma can be overcome by targeting MEK and IGF-1R/PI3K simultaneously [55]. Accordingly, the overexpression of miR-425 inhibits melanoma metastasis, stemness properties and chemoresistance, by repressing the PI3K-Akt pathway by targeting IGF-1 [56]. IGF-1, through the activation of key signaling pathways, enhancing the expression of pro-survival factors, such as Bcl2, Bcl-X(L) and Survivin [57], and favoring DNA damage repair [53], is a key target for enhancing the response of tumor cells to drugs. Our results also indicate that IGF-1 controls many of the mechanisms of chemoresistance in B16-F10 cells, including drug efflux activity through ABC transporter expression and ALDH activity.

IGF-1 blockade could, therefore, effectively interfere with the maintenance of stemness and chemotherapy resistance in melanoma. Moreover, the targeting of IGF-1 might also interfere with the immunomodulatory properties of tumor cells. We previously showed that IGF-1 facilitated immune escape by decreasing the immunogenicity of the murine melanoma B16-F0 cell line [26]. Furthermore, the immunomodulatory properties of tumor cells have recently been linked to EMT and stemness, consistent with a role for CICs in immune escape [15, 58–59]. Indeed, ABCB5^+^ MICs have been shown to induce immune responses less efficiently than the ABCB5^−^ bulk of the tumor [59]. Moreover, melanoma cells programmed to undergo EMT can promote immune escape by generating CD4^+^FOXP3^+^ regulatory T cells and impairing dendritic cell maturation, both *in vitro* and *in vivo* [58].

In conclusion, we provide experimental evidence for a crucial role of IGF-1 in the EMT of B16-F10 cells and in the control of the stem cell phenotype. Collectively, these findings indicate that IGF-1 promotes metastasis not only by favoring proliferation, tumor cell mobility and dissemination, but also by maintaining stemness features crucial for the immune escape, chemoresistance and tumorigenicity of melanoma cells. Our findings identify the IGF/IGFR-1 signaling axis as a key target for the development of anti-tumor treatments against MICs.

## MATERIALS AND METHODS

### Cell lines

The B16-F10 murine melanoma cell line syngeneic to C57BL/6 was obtained from ATCC (Rockville, MD, USA). Clones with IGF-1 downregulation were obtained from wild-type B16-F10 (B16-F10^WT^) after transfection with an episomal IGF-1 antisense vector kindly provided by Dr J. Ilan (Case Western Reserve University, Cleveland, OH, USA). Cells were grown in RPMI 1640 (Gibco BRL, Invitrogen, Cergy-Pontoise, France) supplemented with 10% heat-inactivated fetal bovine serum, 2 mM L-glutamine, 100 U/ml penicillin, 100 mg/ml streptomycin (Gibco) at 37°C, under an atmosphere containing 5% CO_2_. B16-F10 cells were transfected with an empty vector and a clone was selected as the control (B16-F10^CT^). B16-F10^CT^ and IGF-1dull clones were maintained in complete culture medium supplemented with hygromycin B (Invitrogen, Cergy-Pontoise, France).

### Antibodies and reagents

Anti-IGF-1 antibody (1/500, AAM30) was purchased from Bio-Rad AbD Serotec GmbH. Antibodies against MITF (1/100, sc-10999), ZEB1 (1/200, *sc-25388*), p27 (1/200, ref), ABCG2 (1/100, BXP21), cyclin D1 (1/250, sc-450), GAPDH (1/5000, sc25778) and horseradish peroxidase (HRP)-conjugated actin antibody (1/20000, sc-47778 HRP) were obtained from Santa Cruz Biotechnology Inc. (TEBU, *France*). Antibodies specific for N-cadherin (1/1000, 4061), phosphorylated ERK-1/2 (1/1000, 9101), Erk1-2 (1/1000, 4695), and phosphorylated AKT (1/1000, 9271) were obtained from Cell Signaling (Beverly, MA). Antibodies against CD44 (APC, 561862), CD105 (PE, 562759), and CD29 (FITC, 555005) were purchased from BD Biosciences (Oxford, UK). Antibodies against CD133 (PE, 12-1331) and CD24 (PE-Cyanine, 725-0242) were obtained from eBiosciences (Paris, France). Alexa Fluor 488-conjugated phalloidin (1:5000) for F-actin detection was obtained from Invitrogen (Cergy-Pontoise, France). Antibodies against Sox2 (APC, IC2018A), Oct-3/4 (PE, IC1759P), ALDH1A1 (1/250, MAB5869) and IGF-1R (AF-305-NA) and the Ko143 inhibitor (*Tocris* Bioscience, 3241) were purchased from R&D Systems Europe Ltd (Abingdon, UK). The anti-glyceraldehyde 3-phosphate dehydrogenase antibody (1:5000, 381195), and Hoechst 33342 were obtained from Sigma-Aldrich (St Louis, MO). HRP-conjugated (1:5000) and fluorescent (1:200) secondary antibodies were purchased from Jackson ImmunoResearch (West Grove, PA).

### Western blotting

Total cell lysates were prepared by incubation for 30 min on ice in 1% Triton X-100, 20 mM Tris–HCl (pH 7.4), 137 mM NaCl, 2 mM EDTA, 2 mM sodium pyrophosphate, and 10% glycerol in the presence of phosphatase inhibitors (25 mM β-glycerophosphate and 1 mM sodium orthovanadate) and complete protease inhibitor cocktail tablets (Roche Applied Science). The cells were harvested by centrifugation for 5 min at 500 x *g* and lysis of the cell pellet. Cell debris was removed by centrifugation (9000 x *g*), and the proteins in the supernatant were resolved by sodium dodecyl sulfate–polyacrylamide gel electrophoresis and transferred onto a polyvinylidene fluoride membrane. Antibody binding was detected with HRP-conjugated secondary antibodies (1:5000, Jackson Immunoresearch). HRP was detected with an Immobilon Western kit (Millipore, Molsheim, France).

### Scratch wound healing assay

In the scratch wound healing assay, B16-F10WT,B16-F10CT and C10 cells were plated in six-well plates and cultured until they formed confluent monolayers. The cells were then incubated in low-serum content medium for 6 h and wounded in a line across the well with a 200 μl pipette tip. They were washed twice with the low-serum medium to remove debris and non-adherent cells. Time-lapse acquisitions were made with an Axiovert 200 inverted microscope (Zeiss, Oberkochen, Germany) equipped with a controlled-temperature and −CO_2_ chamber. Multiple positions were recorded with an XY motorized stage. Wound closure is presented as the percentage decrease in the freshly wounded area at 0, 6, 12 and 24 h of incubation. The experiment was performed in triplicate.

### Assessment of cell proliferation with the MTT assay

We used the tetrazolium salt 3-(4,5-dimethylthiazol-2-yl)-2,5- diphenyltetrazolium bromide (MTT)) assay, which assesses mitochondrial activity, to evaluate the proliferation of B16-F10^WT^, B16-F10^CT^ and C10 cells *in vitro*. Cells were plated in 96-well plates, at a density of 1 × 10^3^ cells in 200 μL medium per well, and incubated for 96 h. MTT was added to the medium at a concentration of 10 μg/well, and the plates were incubated at 37°C for 4 h. The number of viable cells is directly proportional to the amount of formazan produced. This product was solubilized with 100 μl dimethyl sulfoxide (DMSO), and determined spectrophotometrically at 570 nm, with a microplate reader (Bio-Rad Laboratories, Hercules, CA, USA).

### Clonogenic assay

Cells were used to seed 35 mm petri plates at a clonal density (i.e., 500 cells per well) and were cultured for 10 days. They were then fixed in 4% paraformaldehyde and stained with 0.2% crystal violet solution for the visual observation of colonies. We counted the total number of colonies and the number of colonies with a diameter > 75 μm or containing more than 50 cells.

### Melanosphere formation assays

We assessed the ability of B16-F10^WT^, B16-F10^CT^ and C10 cells to produce melanospheres, by suspending the cells (1 × 10^3^ cells per well) in ultralow attachment six-well plates (Fisher Scientific, Paris, France), in a serum-free medium (SFM) consisting of DMEM/F-12 supplemented with 20 ng/mL EGF (epidermal growth factor; PeproTech, Germany), 20 ng/mL bFGF (basic fibroblast growth factor; PeproTech, Germany), and B27 (1X) (Invitrogen, USA). In all experiments, cells were incubated at 37°C under a humidified atmosphere containing 5% CO_2_, for up to seven days. The total number of melanospheres generated was compared between groups.

### Invasion assay

Cell invasion assays were performed in Transwell chambers (Corning Costar, Cambridge, MA). Matrigel (Becton Dickinson, Bedford, MA) was diluted to a concentration of 5 mg/mL with serum-free RPMI medium, and applied to the polycarbonate membrane filters (8 μm pores) of the chamber for 1 h at 37°C. Cells were placed in the upper chamber, at a density of 5x10^5^ cells/mL in serum-free medium. In the lower chamber, RPMI medium containing 10% FBS was used as a source of chemoattractants. After 8 h of incubation at 37°C, the cells that had not invaded the Matrigel were removed by wiping with a cotton swab, and the cells on the lower surface of the filter were fixed in 100% methanol, washed and stained with DAPI. Quantification was performed under a fluorescence microscope, and the average number of DAPI-stained nuclei per 10x field of view was determined by counting on four randomly selected fields per chamber. Three independent experiments were performed.

### Immunofluorescence

B16-F10^WT^, B16-F10^CT^ and C10 cells were dispensed into an eight-well Lab-Tek tissue culture chamber slide (1 × 10^5^ cells per well; Nunc, Naperville, IL), and intracellular staining (i.e., F-actin, IGF-1, MITF) was performed when the cells reached confluence. The cells were permeabilized by incubation for 5 min with 0.5% Triton X-100 in PBS before incubation with the primary antibody. The cells were washed and incubated for 30 min with Alexa Fluor 488- or 594-conjugated secondary antibodies in 3% bovine serum albumin (BSA) blocking solution (Invitrogen). F-actin organization was revealed by staining the cells with 0.2 μg/mL of rhodamine-conjugated phalloidin for 20 min. The cells were washed twice with PBS, the nuclei were counterstained with DAPI and the cells were mounted in DABCO-glycerol and visualized by confocal fluorescence microscopy (Leica SP5, Solms, Germany).

### Flow cytometry analysis

For the assays described below, we acquired fluorescence data for 10000 to 50000 events on a LSR Fortessa flow cytometer (BD Biosciences, Oxford, UK) and analyzed the data with FACS Diva software (BD Biosciences). Three replicates were used for each condition, and each experiment was performed at least three times.

### Expression of cellular antigens

The expression of cell surface (CD24, CD29, CD44, CD105, CD133) and intracellular (Oct-3/4, SOX2) antigens was analyzed by flow cytometry. Briefly, suspensions of enzymatically detached cells were (for intracytoplasmic staining) or were not (for cell surface staining) permeabilized with BD Transcriptional Factor fix/perm Kit (562574 BD Pharmingen, Le Pont-De-Claix, France), and 10^5^ cells were suspended in staining buffer (0.5% BSA in PBS) and stained with conjugated antibodies directed against the abovementioned cell markers, at the dilutions indicated in the “Antibodies and reagents” section. The cells were then rinsed in phosphate-buffered saline (PBS) and analyzed by flow cytometry.

### Cell cycle analysis

Flow cytometry was used to analyze cell cycle distribution. Cells were treated with trypsin, washed in PBS, fixed at 4°C in 70% cold ethanol and treated with RNase (10 mg/mL) for 30 min at 37°C. DNA content per cell was determined in a LSR Fortessa flow cytometer after staining with propidium iodide (50 μg/mL) for 15 min at room temperature, in the dark. For cell cycle analysis, only signals from single cells were considered (10000 cells/sample).

### ALDH1A1 activity

ALDH1A1 activity was assessed by flow cytometry with an ALDEFLUOR kit (StemCell Technologies, Grenoble, France), in accordance with the manufacturer's instructions. Briefly, 10^6^ cells were incubated for 45 min at 37°C with BODIPY aminoacetaldehyde, which is converted into a fluorescent molecule (BODIPY aminoacetate) by ALDH1A1 in the cytoplasm. The specificity of the fluorescence reaction was demonstrated with the specific ALDH inhibitor diethylaminobenzaldehyde (DEAB). After incubation, the ALDH1A1 activity of the cells was analyzed in a LSR Fortessa flow cytometer. Non-viable cells were excluded from the analysis by propidium iodide staining.

### Dye efflux

The side population (SP) was assessed by staining cells according to Goodell's protocol [60]. Briefly, cells (density 1 × 10^6^/mL) were suspended in prewarmed phenol-free DMEM (Sigma-Aldrich) with 2% FBS. Hoechst 33342 dye (B2261, bisBenzimide HOE 33342; Sigma-Aldrich) was added to a final concentration of 5 μg/mL in the presence or absence of 1 μM Ko143, an ABCG2 extrusion pump inhibitor (Tocris). Cells were incubated at 37°C for 90 min, with intermittent shaking. They were then washed with phenol-free DMEM, centrifuged at 4°C, and resuspended in ice-cold PBS. Propidium iodide (Sigma-Aldrich) was added for the exclusion of dead cells by flow cytometry. The Hoechst 33342 dye was excited at 357 nm and its fluorescence was analyzed at two wavelengths (blue, 402–446 nm; red, 650–670 nm). We evaluated drug efflux activity, by incubating cells suspended in prewarmed DMEM (Sigma-Aldrich) with 2% FBS in polystyrene tubes for 30 min at 37°C with the fluorescent molecule mitoxantrone (0.01 μg/mL; 5 × 105 cells) in the presence or absence of Ko143 (1 μM; Tocris). The cells were then washed, resuspended in prewarmed DMEM with or without Ko143 and incubated for another hour. The accumulation of MIT was assessed by flow cytometry. The mitoxantrone fluorescence emitted was detected at 670/40 nm, after excitation at 633 nm.

### Viability assay

The fluorescent probe fluorescein diacetate (FDA) was used to assess cell viability. Once inside the cell, fluorescein is released by the esterases of living cells, resulting in a measurable fluorescent signal. Cells were used to seed 24-well dishes at 70% confluence and were treated with different concentrations (0.1 to 10 μM) of mitoxantrone for 48 h. They were then incubated for 5 min at 37°C with 0.2 mg/mL FDA, and 10000 cells were analyzed with a LSR Fortessa flow cytometer. Living cells give a positive signal with FDA, whereas dead cells give a negative signal.

### Lung colony formation

B16-F10 is a metastatic cell line that preferentially forms nodules in the lung. The effect of IGF-1 downregulation on B16-F10 cell tumorigenicity was evaluated in C57Bl/6 and NSG mice, by injecting IGF-1dull clones (A6, C10, E11 and C10), B16-F10^WT^ and B16-F10^CT^ cells. Young (5–7 weeks of age) female C57Bl/6 (*n* = 5) and NSG (*n* = 3) mice received intravenous injections (retro-orbital sinus) of 1x10^4^ B16-F10 metastatic melanoma cells. Two weeks later, the mice were killed and their lungs were removed, rinsed in PBS and placed in formalin fixative. The total number of lung nodules was counted under a dissecting microscope for *C57BL/6 mice*. For NSG mice, the burden of nodules in the lung (tumor area/total organ area) was determined quantitatively by manual outlining and analysis with ImageJ software (NIH); the results are expressed as the percentage of the lung area occupied by tumors.

C57Bl/6 and NSG mice (Charles River) were housed in a pathogen-free animal facility under conditions approved by the Animal Care and Use Committee (Comité d'éthique en matière d'expérimentation animale #59, C2EA-59, ‘Paris Centre et Sud’) that approved this study.

### Statistical analysis

Results are expressed as means ± SEM. Statistical analysis was performed by standard two-tailed Student's *t*-test for two groups, in Prism (GraphPad Inc., La Jolla, CA, USA). We considered *p*-values < 0.05, < 0.01 and < 0.001 to be significant, highly significant, and very highly significant, respectively.

## SUPPLEMENTARY MATERIALS


