# Supplementary material for: IGF-1 contributes to the expansion of melanoma-initiating cells through an epithelial-mesenchymal transition process
Source: Oncotarget. 2016 Oct 18;7(50):82511–27. doi: 10.18632/oncotarget.12733 (PMC5347710; doi:10.18632/oncotarget.12733)
Supplement: Supplementary file 1 [file oncotarget-07-82511-s001.pdf]

IGF-1 contributes to the expansion of melanoma-initiating cells through an epithelial-mesenchymal transition process

Supplementary Materials

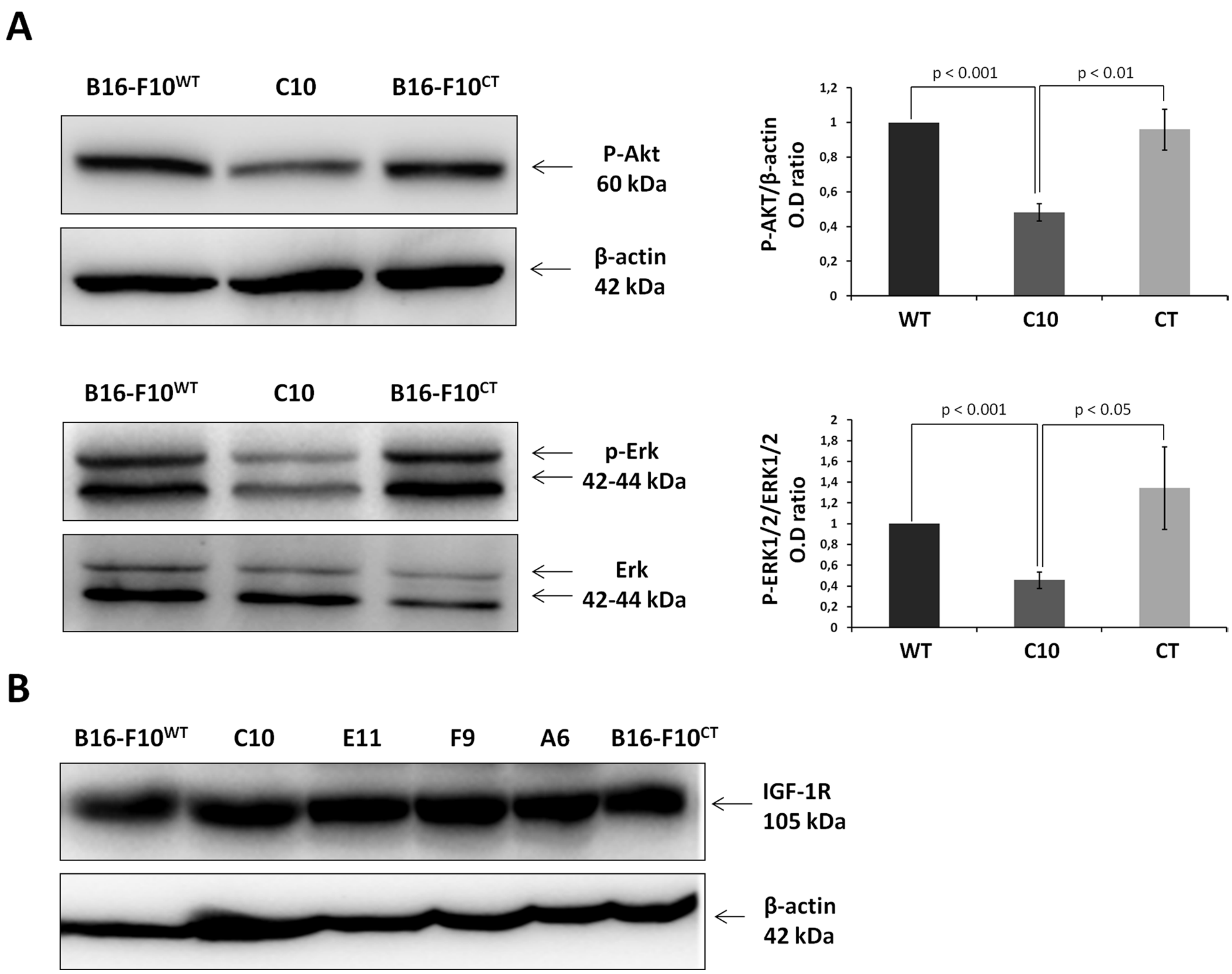

Supplementary Figure S1: IGF-1 signaling pathways are attenuated in the C10 clone. (**A**) Levels of phosphorylated AKT and ERK were analyzed in the C10 clone, B16-F10^{CT} and B16-F10^{WT} cells, by immunoblotting. The phosphorylation signals were quantified as pAKT and pERK versus total β-actin, and ERK. Data are expressed as the mean ± SEM of 4 independent experiments. C10 cells had lower levels of phosphorylated forms of AKT (0.48 ± 0.05 versus 0.96 ± 0.11 and 1 ± 0, $p < 0.05$, $n = 4$) and ERK-1/2 MAPK (0.45 ± 0.07 versus 1.34 ± 0.39 and 1 ± 0, $p < 0.05$, $n = 4$) than B16-F10CT and B16-F10WT cells. (**B**) IGF-1R expression was analyzed in the total lysates of B16-F10WT and B16-F10CT cells and of the four IGF-1dull clones (A6, C10, E11, F9), by western blotting.

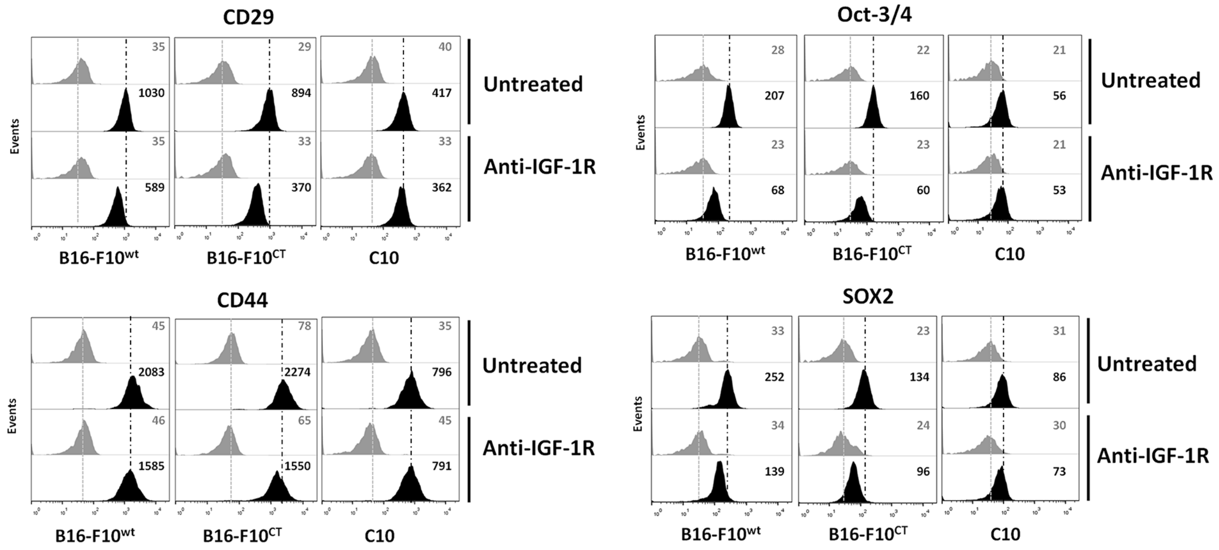

Supplementary Figure S2: IGF-1R neutralization decreases the expression of EMT and stemness markers in B16-F10^{CT} and B16-F10^{WT} cells. Flow cytometry analysis of the CD44 and CD29 mesenchymal markers and of the Oct-3/4 and SOX2 transcription factors in the C10 clone, and in B16-F10^{CT} and B16-F10^{WT} cells with and without neutralizing IGF-1R antibody (0.2 µg/mL) treatment for 48 h.

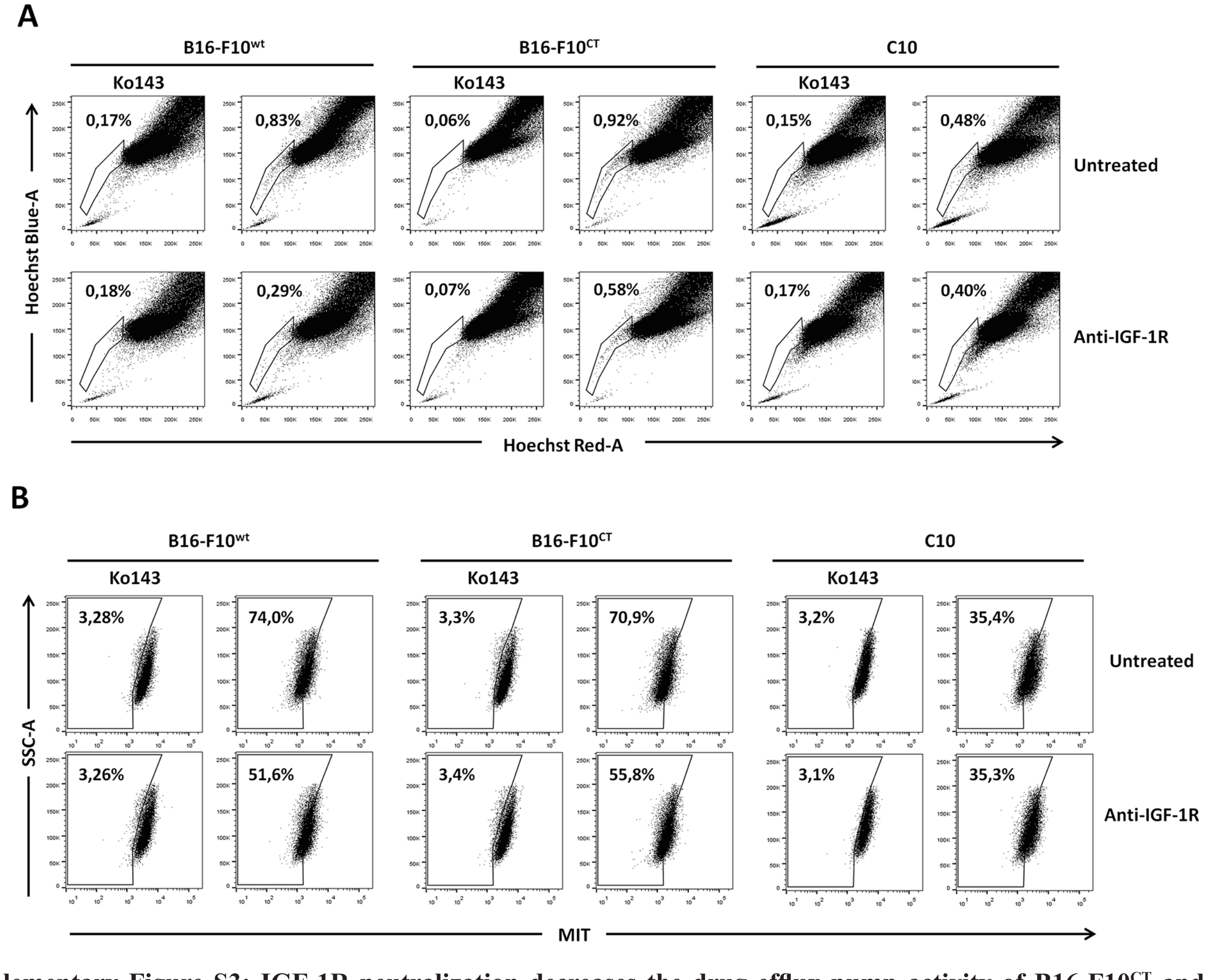

Supplementary Figure S3: IGF-1R neutralization decreases the drug efflux pump activity of B16-F10^{CT} and B16-F10^{WT} cells. (**A**) Side population (SP) analysis. C10, B16-F10^{CT} and B16-F10^{WT} cells with and without neutralizing IGF-1R antibody (0.2 µg/mL) treatment for 48 h were stained with Hoechst 33342 dye in the presence or absence of Ko143 (1 µM) and the SP population was analyzed by flow cytometry. (**B**) Analysis of the intracellular accumulation of MIT in B16-F10^{WT}, B16-F10^{CT} and C10 cells with and without neutralizing IGF-1R antibody treatment for 48 h. Mitoxantrone efflux and ABCG2 activity were determined by flow cytometry, by measuring mitoxantrone accumulation in the presence or absence of the ABCG2 inhibitor Ko143 (1 µM). Percentages of cells displaying MIT efflux are shown at the top left of each histogram.

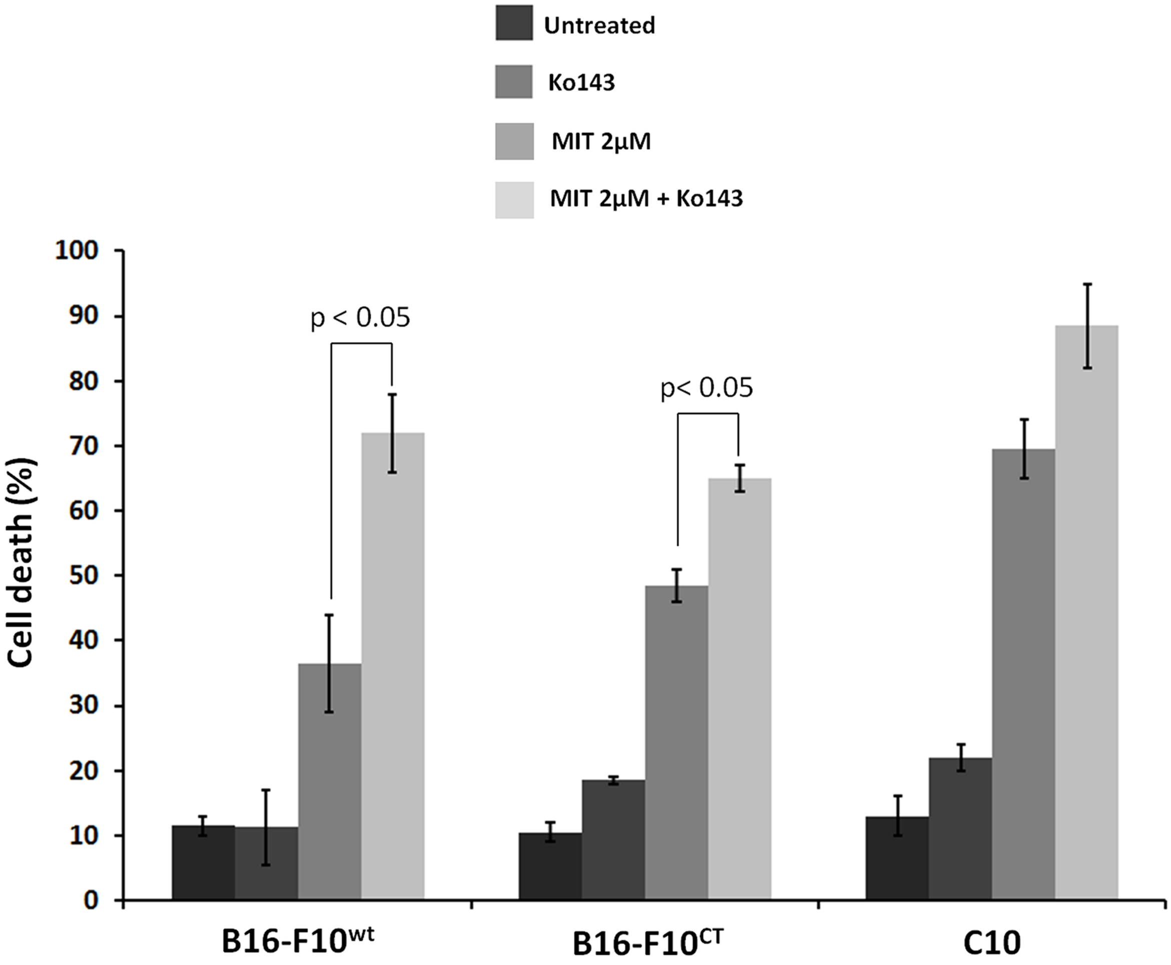

Supplementary Figure S4: ABCG2 protects B16-F10^{WT} and B16-F10^{CT} from MIT-induced cell death. Cells were treated with 2 μM mitoxantrone in the presence or absence of the ABCG2 inhibitor Ko143 (1 μM) and cell viability was determined 48 h later in the FDA assay. Data are expressed as the mean ± SEM. The addition of Ko143 to the culture medium greatly increased cell death in B16-F10CT (48.5 ± 2.5% to 65.0 ± 2.0%, $p < 0.05$, $n = 3$) and B16-F10WT (36.5 ± 7.5% to 72.0 ± 6.0%, $p < 0.05$, $n = 3$) cells treated with 2 μM MIT, whereas it had no significant impact on the death of C10 cells.
